# The Late Jurassic Pterosaur *Rhamphorhynchus*, a Frequent Victim of the Ganoid Fish *Aspidorhynchus*?

**DOI:** 10.1371/journal.pone.0031945

**Published:** 2012-03-07

**Authors:** Eberhard Frey, Helmut Tischlinger

**Affiliations:** 1 Staatliches Museum für Naturkunde Karlsruhe, Karlsruhe, Germany; 2 Co-opted Scientist, Stammham, Germany; University of Western Ontario, Canada

## Abstract

Associations of large vertebrates are exceedingly rare in the Late Jurassic Solnhofen Limestone of Bavaria, Southern Germany. However, there are five specimens of medium-sized pterosaur *Rhamphorhynchus* that lie adjacent to the rostrum of a large individual of the ganoid fish *Aspidorhynchus*. In one of these, a small leptolepidid fish is still sticking in the esophagus of the pterosaur and its stomach is full of fish debris. This suggests that the *Rhamphorhynchus* was seized during or immediately after a successful hunt. According to the fossil record, *Rhamphorhynchus* frequently were accidentally seized by large *Aspidorhnychus*. In some cases the fibrous tissue of the wing membrane got entangled with the rostral teeth such that the fish was unable to get rid of the pterosaur. Such encounters ended fatally for both. Intestinal contents of *Aspidorhynchus*-type fishes are known and mostly comprise fishes and in one single case a *Homoeosaurus*. Obviously *Rhamphorhynchus* did not belong to the prey spectrum of *Aspidorhynchus*.

## Introduction

Pterosaurs, actively flying reptiles of the Mesozoic era, are well documented by an extraordinary fossil record, with the most informative specimens coming from the Middle Jurassic to Early Cretaceous lacustrine deposits of the Jinlingsi and Jehol Group of the western Liaoning Province, China [1]–[4] the Crato and Santana Formations, Chapada do Araripe, Northeast Brazil [5]–[8] and the Late Jurassic Solnhofen Formation, Bavaria, South Germany [9]–[11]. While many soft tissue features of pterosaurs today can be reconstructed to high reliability, food and feeding habits of these animals remain mainly speculative because fossilized intestinal contents are exceedingly rare. Evidence for predators on pterosaurs is restricted to a few remnants in one single pellet [12] or to a few specimens with bite marks [13]–[15]. Here, for the first time, we report on an unusual association of the predatory fish *Aspidorhynchus* and the long-tailed pterosaur *Rhamphorhychus* from the Late Jurassic Solnhofen Limestone (Bavaria, Southern Germany) that proves evidence that *Rhamphorhynchus* fell victim the fish of prey, likely as a result or possibly of a lethal accidental interaction of both animals. At last four additional *Rhamphorhynchus* specimens tightly entangled with the rostrum of a large *Aspidorhynchus* have been discovered but in contrast to the new specimen none of them proves that the *Rhamphorhynchus* was alive when it was seized. Today aerial vertebrate prey, such as birds and bats is recorded for sharks [16], [17] and large actinopterygians [18], however, neither it represents a frequent diet nor is there record of fatal encounters for both victim and predator in the scientific literature.

While much is known on the skeletal and soft tissue anatomy of pterosaurs evidence for their position in the Mesozoic food chains is exceedingly sparse in the fossil record. Remnants of fishes inside the ribcage of a pterosaur have been reported for a single specimen of *Eudimorphodon ranzii* (Middle Triassic, Norian, Northern Italy) [19]. According to Wild [19] the fish remains refer to *Parapholidophorus*, which abundantly occurs in the *Eudimorphodon* bearing beds. Two specimens of *Rhamphorhynchus muensteri* (Late Jurassic, Tithonian, Solnhofen Limestone, Southern Germany) contain remnants of small leptolepidid fishes [9], [10], [20]. Fragments of undetermined fishes and crustaceans being preserved in the throat area of a *Pteranodon* (Late Cretaceous, Santonian, North America) and fish debris in the gular area of a *Pterodactylus* (Late Jurassic, Tithonian, Solnhofen Limestone, Southern Germany) respectively have been tentatively interpreted as the contents of a throat pouch [21], [22]. One *Preondactylus* specimen from the Middle Triassic of Northern Italy is preserved as a crushed agglomeration of bone that likely formed the regurgitation pellet produced by a large fish [12].

## Results

**Figure 1:**
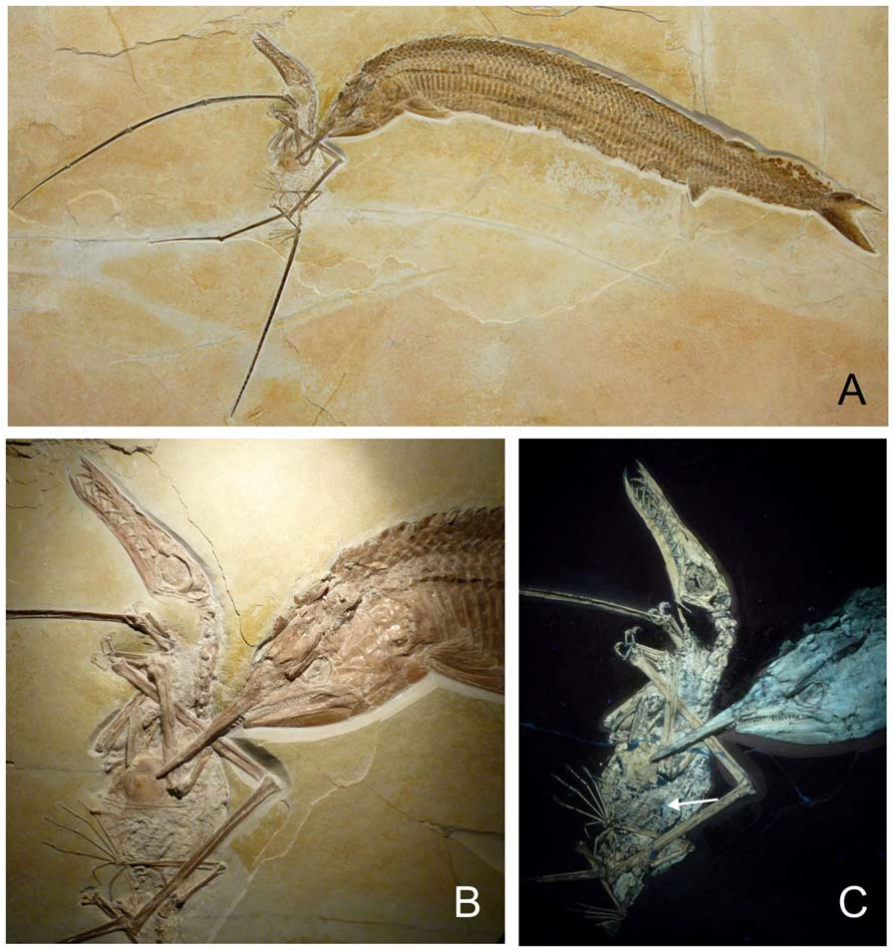
Fossilized hunting scene; A) specimen WDC CSG 255: an *Aspidorhynchus* and a *Rhamphorhynchus* in fatal encounter, B) section of the specimen showing the way both animals are entangled. Note that the jaws of the *Aspidorhynchus* did not get hold of a bone, but apparently of the flight membrane of the left wing. The distorted left wing finger is suggestive for this. C) A similar section under UV light, the arrow marks intestinal contents consisting of digested fish remains.

**Figure 2:**
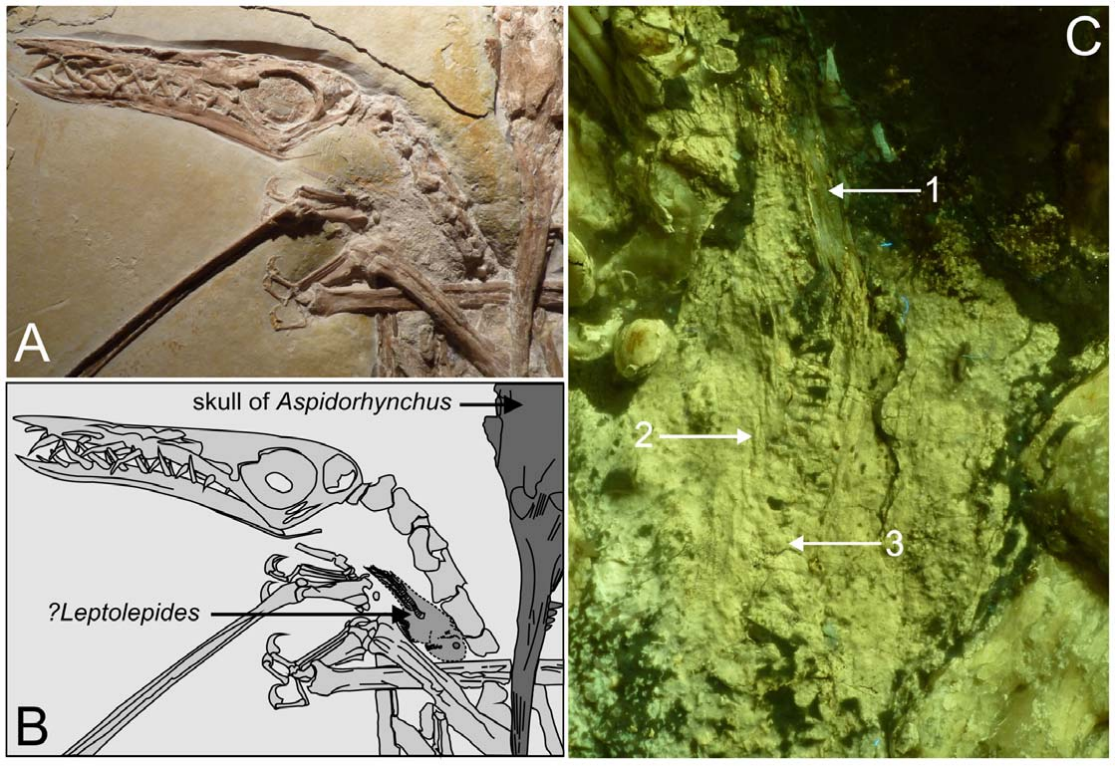
Predator and prey; the final meal of a *Rhamphorhynchus* is still sticking in the pharynx. The straight vertebral column and the closed tail fin, which is orientated towards the mouth cavity suggests that the *?Leptolepides* was not regurgitated during the agony of the pterosaur. Furthermore the prey does not show any trace of digestion. A) photograph of the specimen WDC CSG 255 showing the position of the *?Leptolepides* in the throat of the *Rhamyphorhychus*. To the right hand side the skull of the *Aspidorhynchus* is visible. B) line drawing of (A), C) close-up of *?Leptolepides* under filtered UV light: 1 caudal fin, 2 neural spines, 3 vertebral column.

The Solnhofen limestone has yielded a variety of vertebrate fossils in an excellent state of preservation [23]. However, a close association of large vertebrates is exceedingly rare. An outstanding exception here are four slabs with the predatory fish *Aspidorhynchus acutirostris* closely associated with *Rhamphorhynchus muensteri*. In three of these slabs the virtually complete skeleton of a subadult or adult *Rhamphorhynchus* lies immediately adjacent to the jaws of the *Aspidorhynchus* (Figure 1). In one of the slabs a wing bone of the *Rhamphorhynchus* is sticking between the jaws of the fish (Figure 1 B). All four slabs have in common that the skull of the *Aspidorhynchus* lies adjacent to one of the wing bones of the *Rhamphorhynchus* specimen suggesting a contact interaction between the mouth of the fish and at least the wing membrane of the pterosaur. However, none of these specimens tells us, whether or not the *Rhamphorhynchus* was alive prior to being seized by the *Aspidorhynchus*. There is only one single record of two *Rhamphorhynchus* from the Solnhofen Formation being preserved in one slab in body to body contact [24]. Large *Aspidorhynchus* until now have never been found associated with other fossils other than *Rhamphorhynchus*. The specimen we describe here for the first time is housed in the Wyoming Dinosaur Center (WDC), Thermopolis, U.S.A. under the collection number WDC CSG 255. The specimen was found in the year 2009 in a plattenkalk quarry NW of the town of Eichstätt (Bavaria, Southern Germany) within the Solnhofen Lithographic Limestone (*Hybonotum* Zone, Riedense Subzone), [25]. Apparently the *Rhamphorhynchus* had just caught a small fish and was about to swallow it head first, when the *Aspidorhynchus* attacked. The fish tail yet sticking in the pharyngeal region of the throat and the excellent preservation of the tiny fish without any trace of digestion suggests that swallowing was not completed and that the *Rhamphorhynchus* was alive and airborne during the attack. A regurgitation of the leptolepidid fish tail first into the pharyngeal region as a result of agony of the pterosaur would not have been possible, because the fins and the opercula of the fish would have braced themselves against the wall of the narrow esophagus. Instead, the fins of the fish lay smoothly alongside the body ande the lepidotrichs of the caudal fin are folded together and lay perfectly straight because the caudal fin apparently had just passed the larynx of the *Rhamphorhynchus*. Furthermore, there is no evidence that the half digested fish debris in the abdominal area (Figure 2 C) has been regurgitated into the esophagus.

The *Aspidorhynchus* apparently attacked from in front when the *Rhamphorhynchus* still flew low above water surface, grabbed the left wing level with the distal end of the antebrachium close to the carpus and pulled the pterosaur under water. While the *Rhamphorhynchus* rapidly drowned with its last prey in the throat the cause of death of the *Aspidorhynchus* remains speculative. Evidently, the fish could not swallow the pterosaur due to its size and bulky skeleton. Furthermore, ganoid fishes like *Aspidorhynchus* have skulls with limited kinetic options such that they were not suitable to manipulate prey that exceeded the standard gape of the jaws. Obviously, the fish was neither able to swallow the pterosaur, neither was it able to get rid of its oversized victim. Possibly the aktinofibrils of the tough and leathery wing membrane of the pterosaur [26]–[28] got jammed between the densely packed teeth of the fish. Like most extant fish *Aspidorhynchus* had no other possibility to get rid of its unwanted victim than trying to shake it loose or swimming rapid spinning or twisting maneuvers. That the fish in fact tried to get rid of the pterosaur by vigorous movements of its head is evidenced by the distortion of the left wing finger elements, while the remaining skeleton of the *Rhamphorhynchus* lies in natural articulation. Apparently, the flight membrane tissue remained jammed between the teeth, while the interphalangeal ligaments of the left wing finger ruptured under the power of the fish tearing at the flight membrane. Finally, the entire wing finger of the drowned pterosaur was pulled under the antebrachium. Such a distortion can only happen when the proximal part of the flight membrane, likely the thin and structurally weak tenopatagium, [29], was dramatically overextended or even had ruptured. The most likely scenario is that the *Aspidorhynchus* fought its victim for a period of time, thereby rapidly sinking into the hostile anoxic water layer of the Late Jurassic Eichstätt basin [30]–[32], where it was instantly suffocated. Still linked together, both carcasses sank to the sea floor, whereby the pterosaur contacted the ground first, likely being pushed down by the massive head of the *Aspidorhynchus*.

## Discussion

The specimen presented here firstly proves evidence that the Late Jurassic pterosaurs of the genus *Rhamphorhynchus* actively hunted fish by grabbing or skimming them out of the water thereby closely approaching the water surface due to their short necks. Skimming as hunting behavior of *Rhamphorhynchus* has been previously suggested [10]–[20] and would have been mechanically possible thanks to the short neck reinforced by cervical ribs and a blade-like dorsally curved mandibular rostrum. Skimming as a hunting strategy was later also assumed for the late Early Cretaceous azhdarchoid pterosaur *Thalassodromeus sethi* [33], but in this case the mechanical prerequisites concerning the stability of the neck and mandibular lever cast some doubt on this idea [34], [35], [37].

A second conclusion refers to the fact that *Aspidorhynchus* at least occasionally hunted close to the water surface, where schools of small leptolepidid fishes must have lived. Either, *Aspidorhynchus* could spot potential prey not only near but also above water surface relying on its eyesight as do some extant fishes like e.g. *Toxotes* and *Osteoglossus* [36], [18] or reacted to the turbulences caused by the pterosaur, when its mandibular rostrum skimmed through the water during hunting. In all specimens of *Aspidorhynchus* with an associated *Rhamphorhynchus* the fish was anatomically unable to swallow the pterosaur. In these cases the attack was a lethal error on the side of *Aspidorhynchus*. Such errors are frequently reported in the fossil record, but to our knowledge are predominantly restricted to oversized prey fishes, where the opercula of the prey fish mostly entangle with the gill rakers and arches of the predator during the effort to regurgitate the prey [37], [38].

Despite four specimens of large *Aspidorhnychus* are associated with a *Rhamphorhynchus* adjacent to the jaws there is no evidence that pterosaurs belonged to the prey spectrum of *Aspidorhynchus*. The suggestion that the restricted cranial kinetics of the fish limited the size of the prey is strongly supported by the intestinal contents known from fishes of the aspidorhynchid morphotype. The effect of the limited cranial kinesis of aspidorhynchid actinopterygians with respect to the maximum prey size is demonstrated by a 0.6 meter long *Aspidorhynchus* that had tried to swallow a 0.2 to 0.25 meter long *Pholidophorus* head first [37]. The caudal half of the fusiform pachycormid fish *Pholidophorus* is still outside the mouth of the *Aspidorhynchus* suggesting that a potential prey of the latter must have had a diameter of 50 mm, likely less. Several *Aspidorhynchus* bear remnants of much smaller fishes in their intestinal tracts and one *Belonostomus* is known that had ingested a *Homoeosaurus* [39], [40], ([41] (p. 98, fig. 97). Not a single fragment of a pterosaur had been discovered inside an *Aspidorhynchus* to date, which would have been the case, if the fish potentially was able to swallow a pterosaur.

*Rhamphorhynchus* principally had two options to seize dead or alive fishes out of the water: grabbing or skimming [10]. For grabbing a fish the time of low water surface approach is short and therefore an interaction of a *Rhamphorhynchus* with any aquatic predator is unlikely. Skimming, in contrast, took time and resulted in a significant signal of turbulence, when the mandibular rostrum ploughed through the silent water surface. Such turbulences attract all kinds of fishes and are also were easily detectable for an *Aspidorhynchus*. Furthermore, the vane at the terminus of the long tail of the pterosaur could have contacted the water surface too due to the extremely low surface approach with a flight altitude of no more than 50 mm. Large *Aspidorhynchus* thus could grab a skimming *Rhamphorhynchus* by just raising the head through the water surface. The specimen presented here strongly suggests that *Aspidorhynchus* actually did exactly this.

## Materials and Methods

As a rule skeletal remains of fossils and slightly mineralized soft parts from the Late Jurassic plattenkalks of southern Germany are fluorescent under ultraviolet radiation. Many times details of skeletal remains as well as soft parts can be more precisely investigated in ultraviolet light than in visible light. Delicate skeletal elements including different bony components and remains of soft parts sometimes are poorly or not discernable in visible light but illuminate conspicuously under filtered UV. Each limestone slab and bone or tissue will react differently to different light wave lengths and has to be captured differently with varying exposure times and filters. Therefore, a correct combination is needed to highlight the area of interest [42], [43].

For the ultraviolet-light investigation of the specimen we used a set of UVA lamps with a wavelength of 365 to 366 nanometers and an intensity between 4000 and more than 50000 microwatts per 10 mm^2^, depending on the distance concerned and the number of lamps. The application of different filters allows a selective visualisation of peculiar fine structures. For this, a set of different color correction filters was necessary. The optimum filtering was tested in a series of experiments. In addition the number and combination of filters were subject to the displacement, intensity, and incident angle of the ultraviolet lamps. The UV photos were taken with a Panasonic DMC digital bridge camera, equipped with a macro lens (Raynox M-250).
